# Harnessing the power of mobile and messaging apps for risk communication and intervention during the COVID-19 pandemic: lessons from the Western Pacific

**DOI:** 10.5365/wpsar.2024.15.3.1156

**Published:** 2024-08-20

**Authors:** Fernan Talamayan, Lieke Visser, Babatunde Olowokure, Nancy Wong, Wenyajing Zhang

**Affiliations:** a Health Emergencies Programme, World Health Organization Regional Office for the Western Pacific, Manila, Philippines.

## Abstract

**Problem:**

The spread of mis- and disinformation on mobile and messaging apps during the COVID-19 pandemic not only fuelled anxieties and mistrust in health authorities but also undermined the effectiveness of the overall public health response.

**Context:**

Mobile and messaging apps help users stay informed and connected to their families, friends, colleagues and communities. However, during the COVID-19 pandemic, these apps were also one of the primary channels where mis- and disinformation were circulated.

**Action:**

Recognizing the importance of including mobile and messaging apps in risk communication and emergency response strategies, the World Health Organization (WHO) and some countries in the WHO Western Pacific Region independently piloted initiatives to reach messaging app users, meet their evolving information needs, and streamline health ministry communication.

**Outcome:**

The enhanced use of mobile and messaging apps enabled consistent and timely communication and improved coordination during the COVID-19 pandemic. Leveraging their features also helped identify and potentially fill crucial information gaps, mitigating the harms of mis- and disinformation and fostering stronger trust in health authorities.

**Discussion:**

The findings from the work carried out by WHO and countries in the Western Pacific Region identified some promising innovative communication interventions using mobile and messaging apps. While these interventions should be further explored and evaluated, they have demonstrated that interventions need to be proactive, flexible, and able to adapt to changes in mis- and disinformation content being shared through messaging apps.

## PROBLEM

The COVID-19 pandemic saw the spread of health-related mis- and disinformation on popular social networking messaging apps, such as WhatsApp, Facebook Messenger, Telegram and Viber, among others. (*1*) While these apps enable connections and the exchange of important health information, the relationships fostered between users of these platforms have also increased their susceptibility to consuming and sharing mis- and disinformation. (*2*) In closed social networks, characterized by intimacy and trust among contacts who generally know one another, there is a greater possibility of content being believed and shared without adequate fact-checking. (*3*)

In an era of expanding digital connectivity, the rapid spread of information can be both beneficial and detrimental to public health: it is useful for swiftly responding to people's concerns and filling information voids, yet it can also amplify harmful messages. The widespread circulation of mis- and disinformation exacerbates disease- or pandemic-related challenges by fuelling fears, encouraging negative health behaviours, eroding trust in health authorities and undermining the effectiveness of public health and social measures. (*4*)

This work is an effort to understand communication using private messaging apps during the COVID-19 pandemic as this may help to improve public health messaging, including addressing mis- and disinformation, in future public health emergencies.

## CONTEXT

Messaging apps such as Facebook Messenger, WeChat, WhatsApp and Telegram account for approximately  5 billion users globally. (*5*) They have extensive reach, and according to a report by the digital analysis firm Kepios, in 2023, Facebook Messenger had the majority share of the population aged 13 and above in several countries in the Western Pacific, including Mongolia (79.8%), Viet Nam (66.3%), Tonga (66.2%), New Zealand (60.6%), the Philippines (60.5%) and Fiji (59.6%). (*6*) The same report highlighted that 71.1% of active global Facebook/Meta users aged 16–64 years use the platform for messaging family and friends. This figure reflects users' gravitation towards free apps that support private, personal communication. The design of most messaging apps is influenced by these specific user preferences, offering a range of features from secure messaging to group chats and multimedia sharing.

Despite the popularity and practical benefits of messaging apps, WHO recognized and highlighted their role as disseminators of rumours and mis- and disinformation, especially during the COVID-19 pandemic. (*7*) In Singapore, it has been reported that Telegram has become a source for the circulation of misinformation through chat groups that have attracted a large following. (*8*) Members of these groups questioned the safety and effectiveness of mRNA COVID-19 vaccines and prompted others to explore the use of ivermectin for curing COVID-19. (*9*) Viber chat groups have played a significant role in the spread of COVID-19 misinformation in the Philippines, causing panic among citizens and leading to potential superspreader events. (*10*) In Australia, a study revealed that individuals who endorsed COVID-19 misinformation on social media exhibited lower levels of confidence in government, trust in scientific institutions, perceived COVID-19 threat and digital health literacy. (*11*)

## ACTION

With increasing numbers of people turning to social media for news and information, achieving maximum reach, engagement and impact necessitated the adoption of innovative communication strategies and interventions. WHO and countries in the WHO Western Pacific Region independently explored various approaches to fill information voids and reduce the spread of mis- and disinformation, ranging from creating shareable content to engaging key influencers. They also piloted initiatives to use platform features in their emergency response and connect with mobile and messaging app users through broadcast channels and interactive chatbots (that is, computer programmes that simulate conversation with human users to provide them with the information or assistance they need). (*12*)

For instance, Singapore's Ministry of Communications and Information launched broadcast channels on Telegram and X (previously Twitter) to complement their existing Facebook, Instagram and WhatsApp channels and offer more options for the public to obtain information on COVID-19. (*13*) In early 2020, the Government of Australia launched a COVID-19 WhatsApp bot (decommissioned in 2022). (*14*) The Philippine Department of Health also created a chatbot service called Knowledge Informs Responsible Action (KIRA), which is accessible through popular messaging apps such as Facebook Messenger and Viber. (*15*) At the height of the COVID-19 pandemic, these apps had the capacity to spread messages with great velocity due to the millions of members.

The pandemic prompted the development of new chatbot features and functions to understand and meet people's evolving needs. For example, KIRA enabled the Philippine Department of Health to capture public sentiment towards COVID-19 vaccination, track citizens’ satisfaction with their vaccination programme and receive reports on online mis- and disinformation. This approach enabled health authorities to disseminate relevant health information and implement evidence-based COVID-19 responses. Meanwhile, the COVID-19 WhatsApp bot in Australia had a registration feature specifically for self-isolation. (*14*) Another example is Viet Nam's local messaging app Zalo, which offered users affected by COVID-19 the option to seek or provide emergency support and receive remote medical advice in Ho Chi Minh City, Đồng Nai, Bình Dương and Long An. (*16*) Overall, these initiatives showcase the adaptability of messaging apps in addressing varied public health needs.

In addition to collecting data and disseminating health information, messaging apps were also used to streamline ministry communication and decision-making. For example, in Cambodia, the Ministry of Health informed the authors that Telegram was adopted as a tool to improve coordination among various government agencies.

At the regional level, WHO conducted preliminary research in 2021 to determine ways that ministries and institutions could mitigate the potential harms of the unchecked spread of COVID-19 and vaccine-related mis- and disinformation. Insights from the research were used to design and implement capacity-building initiatives that could reinforce the use of mobile and messaging apps for combating mis- and disinformation.

## OUTCOME

The spread of mis- and disinformation online pointed to the need for better integration of digital platforms, especially mobile and messaging apps, into the planning and operationalization of public health strategies. Several lessons were identified from the COVID-19 experience, including the effectiveness of mobile and messaging apps in streamlining the COVID-19 response, delivering accurate health information, mitigating the harmful effects of mis- and disinformation and coordinating inter-agency communications.

Messaging apps also played a role in improving people's well-being and building greater trust in health authorities. A study of the psychological well-being of 1145 adults in Singapore revealed that exposure to government WhatsApp messages helped to reduce depressive symptoms associated with receiving COVID-19 updates, while increased trust in official WhatsApp messages helped to decrease people's anxiety during the pandemic. (*17*) Singapore's successful use of messaging apps to disseminate information during the pandemic highlighted the effectiveness of a multiplatform, multilanguage and multiformat approach in providing reliable and timely updates to the public. The strategy not only provided the public with accurate and up-to-date information but also fostered trust between the government and the population. (*18*)

Embracing the features of mobile and messaging apps helped enhance response capabilities and interaction with health authorities' target audience. In January 2022, Viet Nam's Ministry of Information and Communication reported that over 100 000 people received aid through Zalo's Connect feature. (*19*) KIRA's July 2022 statistical analysis revealed that their content had been used by 1 million users from over 1400 local governments in the Philippines, resulting in almost 35 million interactions. (*20*) Through KIRA, messaging app users were able to input their questions and receive responses, functioning as hotlines akin to online customer service provided by private companies.

The Cambodian Ministry of Health informed the authors that Telegram promoted swift inter-agency coordination during the COVID-19 pandemic. The app was used to share critical information and guidance and flag emerging mis- and disinformation. This strategy enabled the development of consistent messaging and quick action against mis- and disinformation. However, despite the app's advantages, one challenge that emerged was maintaining comprehensive records of these communications. The absence of proper archiving in messaging apps makes it difficult to retrieve official communication or verify previously discussed information. These apps often support various media types such as text, images, audio and video. Thus, it is problematic to track and organize diverse data formats into coherent records.

Despite the challenges revealed by WHO and countries in the Western Pacific Region, experience and lessons learnt demonstrated the importance of disseminating clear, timely and consistent health messages to effectively address questions about COVID-19 and vaccines.

## Discussion

Various actions taken by WHO and countries in the Western Pacific Region during the COVID-19 pandemic offer valuable insights into both the potential and challenges of leveraging mobile and messaging apps for risk communication and health emergency response. Singapore's multiplatform strategy, Australia's WhatsApp bot and Viet Nam's Zalo app demonstrated innovative uses of technology to meet specific local needs, showcasing adaptability and responsiveness. Integrating the KIRA chatbot into popular messaging apps in the Philippines enhanced the Department of Health's ability to listen and respond to people's questions and evolving needs. (*15*) The use of messaging apps for inter-agency coordination, as observed in Cambodia, also highlighted their versatility beyond their conventional functions.

This trend could also be observed globally. International organizations such as WHO and the United Nations Children's Fund (UNICEF) spearheaded online initiatives to improve global understanding of COVID-19. WHO rolled out the WHO Health Alert Chatbot on WhatsApp and Facebook in early 2020, offering information on the latest COVID-19 figures and cases, guidelines for self-protection, frequently asked questions, travel advisories, news and press releases in different formats. (*21*) UNICEF launched the U-Report COVID-19 Information Chatbot in the same year to enhance risk communication and community engagement around COVID-19. (*22*) Information in both chatbots is available in multiple languages. This holds immense significance as it ensures inclusivity and accessibility for messaging app users, transcends language barriers and empowers individuals to access accurate COVID-19 information in the language with which they are most comfortable.

Despite the benefits of integrating mobile and messaging apps into various risk communication strategies, there are significant challenges, particularly in maintaining people's trust and protecting their privacy during data collection processes. Mobile and messaging apps are, in most cases, encrypted to protect the data and content of their users. For health authorities, this means data are unavailable to identify people's information needs. There is also concern over authorities’ potential privacy breaches. (*23*) Consent is not regularly obtained for messaging apps, and confidentiality, privacy, data security and record-keeping remain areas of concern. (*24*)

In summary, by leveraging messaging app features such as broadcast messages and chatbots, various countries were able to provide messaging app users access to accurate health information and an avenue to inform ministries of their needs. The rapid circulation of accurate and well coordinated health messages on messaging apps helped achieve two things: pre-empt information voids that could be filled with mis- and disinformation; and foster trust in health authorities. Examples from the Western Pacific Region demonstrate the value of exploring how these pilots could be applied to a wider range of health communication strategies, extending beyond emergency response. Given that our results are based on a small subset of the overall conversations on mobile and messaging apps, these interventions should be further evaluated to determine which approaches, or combination of approaches, may be most effective.
